# Supplementary material for: In-vivo efficacy of biodegradable ultrahigh ductility Mg-Li-Zn alloy tracheal stents for pediatric airway obstruction
Source: Commun Biol. 2020 Dec 18;3:787. doi: 10.1038/s42003-020-01400-7 (PMC7749127; doi:10.1038/s42003-020-01400-7)
Supplement: Supplementary file 1 — Supplementary Information [file 42003_2020_1400_MOESM1_ESM.pdf]

In-vivo efficacy of biodegradable ultra-high ductility Mg-Li-Zn alloy tracheal stents for pediatric airway obstruction

Jingyao Wu[a], Leila J. Mady[b], Abhijit Roy[a], Ali Mübin Aral[c], Boeun Lee[a], Feng Zheng[d], Toma Catalin[e,f], Youngjae Chun[a, f, g], William R. Wagner[a, c, f, h,] , Ke Yang[d], Humberto E. Trejo Bittar[i], David Chi[j], Prashant N. Kumta[a, f, h, k, l,m]*

[a] Department of Bioengineering, University of Pittsburgh, Pittsburgh, PA 15261, USA

[b] Department of Otolaryngology, University of Pittsburgh, Pittsburgh, PA 15261, USA

[c] Department of Surgery, University of Pittsburgh, Pittsburgh, PA 15261, USA

[d] Institute of Metal Research, Chinese Academic of Sciences, Shenyang, 110016, China

[e] Heart and Vascular Institute, University of Pittsburgh Medical Center, Pittsburgh, PA 15261, USA.

[f] McGowan Institute of Regenerative Medicine, Pittsburgh, PA 15261, USA

[g] Department of Industrial Engineering, University of Pittsburgh, Pittsburgh, PA 15261, USA

[h] Department of Chemical and Petroleum Engineering, University of Pittsburgh, Pittsburgh, PA 15261, USA

[i] Department of Pathology, University of Pittsburgh Medical Center, Pittsburgh, PA 15213, USA

[j] Department of Otolaryngology, Children's Hospital of Pittsburgh of UPMC, Pittsburgh, PA 15224, USA.

[k] Department of Mechanical Engineering and Materials Science, University of Pittsburgh, Pittsburgh, PA 15261, USA

[l] Department of Oral Biology, School of Dental Medicine, University of Pittsburgh, Pittsburgh, PA 15261, USA

[m] Center for Complex Engineered Multi-functional Materials, University of Pittsburgh, Pittsburgh, PA 15261,

* Corresponding author: Tel.: +1-412-648-0223; fax: +1-412-624-3699

E-mail address: pkumta@pitt.edu

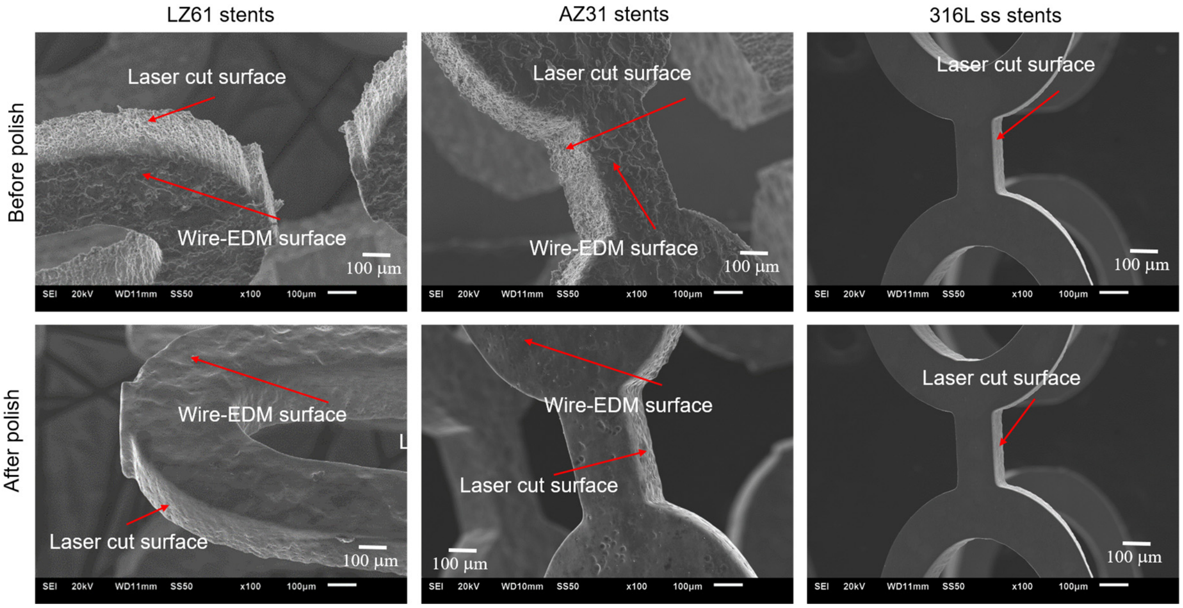

Supplementary Figure 1. Surface morphology of LZ61-KBMS alloy based stents, AZ31 alloy based stents and 316L SS stent before and after electrochemical polishing. For 316L SS stent, since the tube was directly purchased from Goodfellow(Coraopolis, PA), the external surface was smoother than the wire-EDM machined LZ61-KBMS and AZ31 alloy based stents.

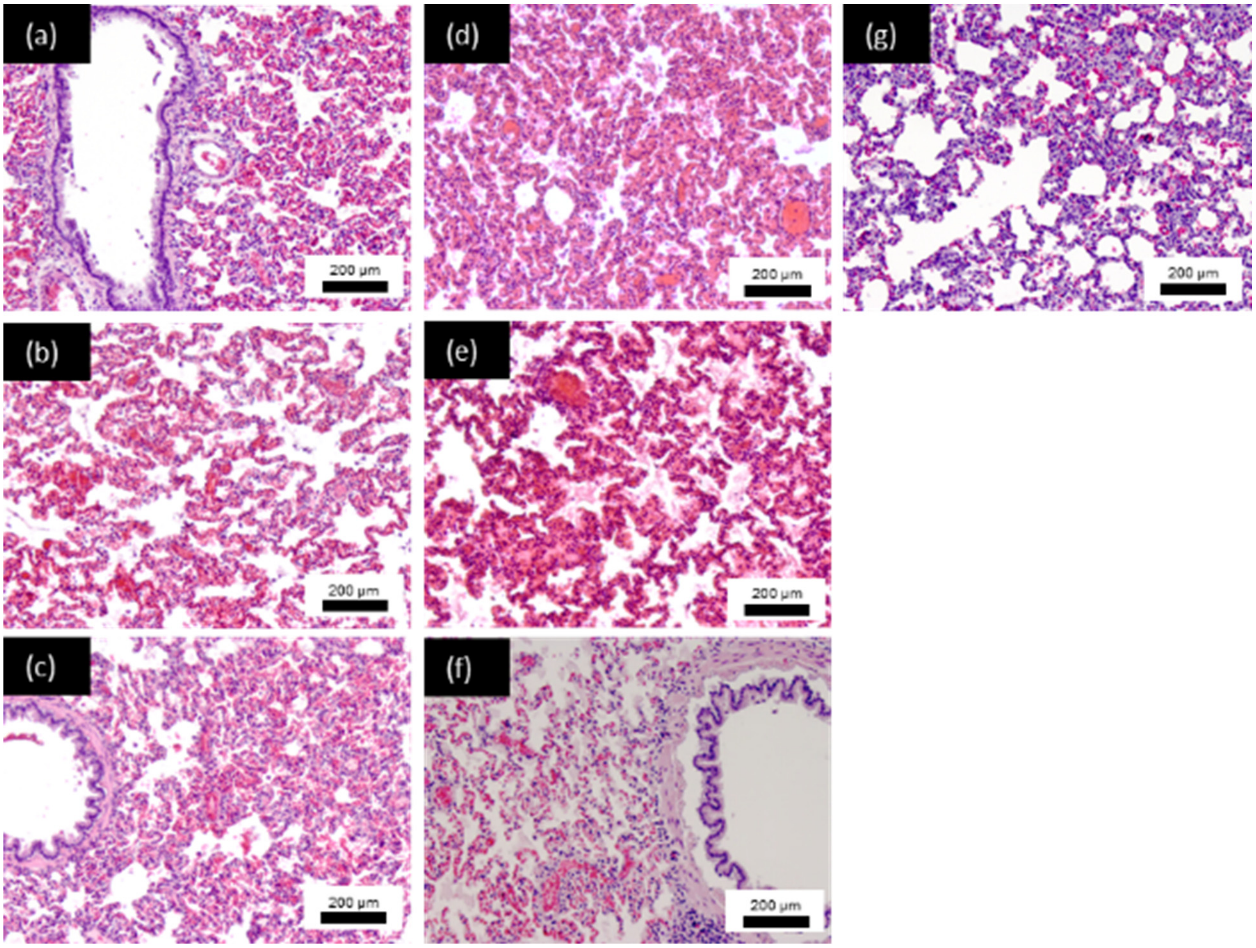

Supplementary Figure 2. H&E staining of the lung tissue. LZ61-KBMS stent group at (a) 4 weeks, (b) 8 weeks and (c) 12 weeks post implantation; 316L SS stent group at (d) 4 weeks, (e) 8 weeks and (f) 12 weeks post implantation; (g) normal rabbit lung tissue.

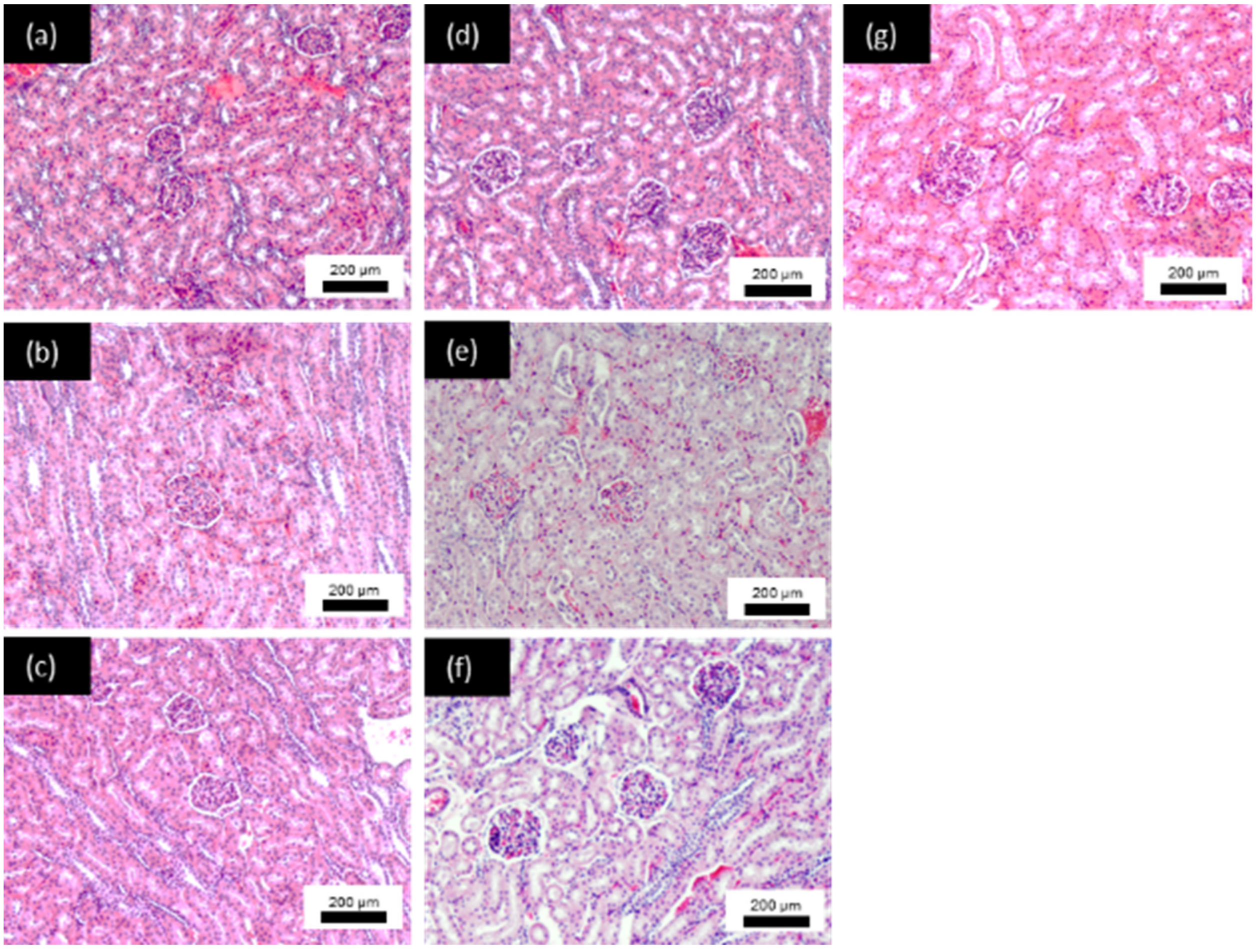

Supplementary Figure 3. H&E staining of the kidney tissue. LZ61-KBMS stent group at (a) 4 weeks, (b) 8 weeks and (c) 12 weeks post implantation; 316L SS stent group at (d) 4 weeks, (e) 8 weeks and (f) 12 weeks post implantation; (g) normal rabbit kidney tissue.

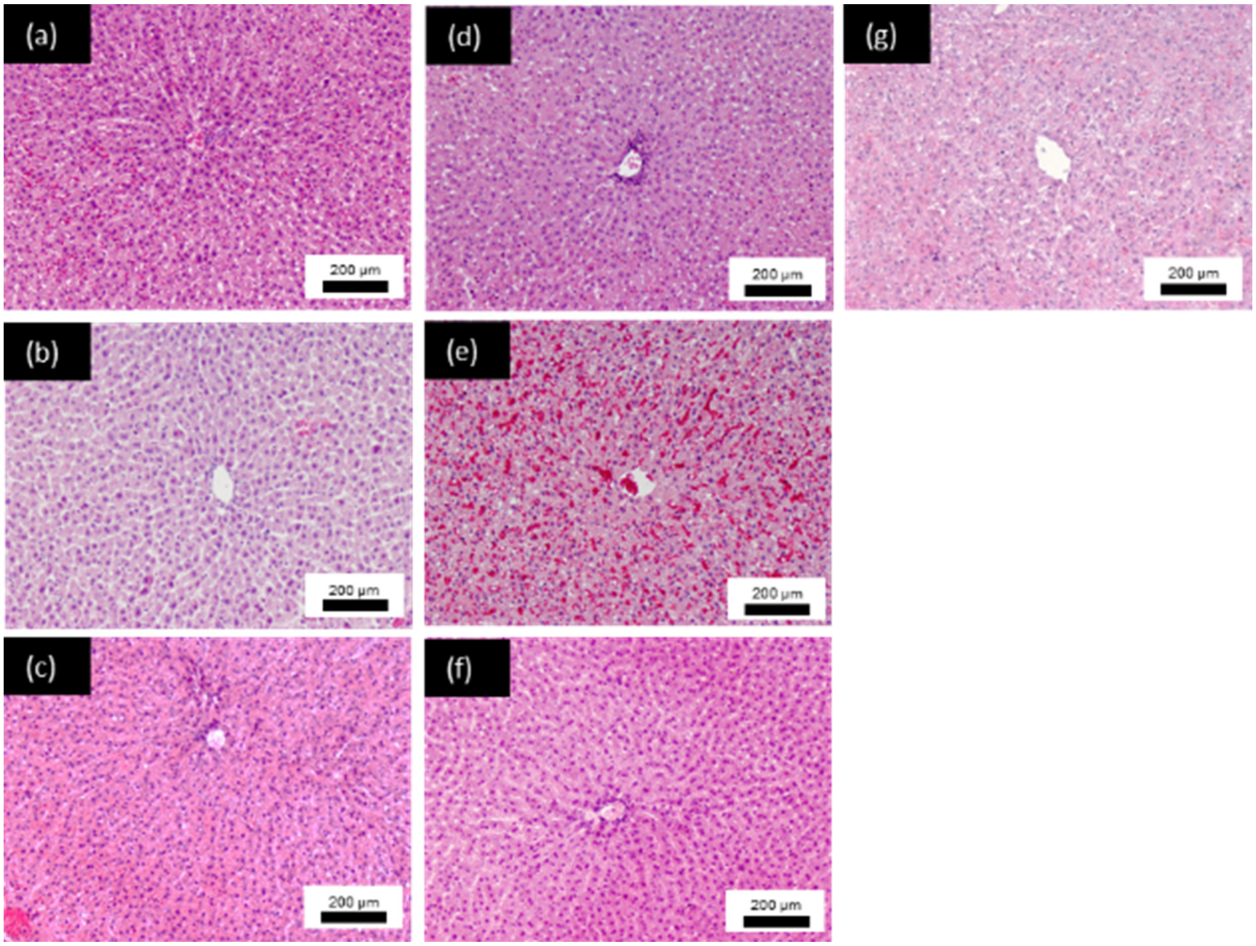

Supplementary Figure 4. H&E staining of the liver tissue. LZ61-KBMS stent group at (a) 4 weeks, (b) 8 weeks and (c) 12 weeks post implantation; 316L SS stent group at (d) 4 weeks, (e) 8 weeks and (f) 12 weeks post implantation; (g) normal rabbit liver tissue.

Supplementary Table 1 The chemical composition of the LZ61-KBMS alloy.

Chemical composition (wt. %)	Mg-6Li-1Zn
Li	6.11±0.13
Al	0.04±0.06
Zn	0.92±0.08
Mn	0.00835±0.00013
Fe	0.00045±0.0004
Cu	0.00040±0.00001
Ni	0.00018±0.000003

Supplementary Table 2 The composition of the electrolyte used for electrochemical polishing of different tracheal stents.

	**Component**	**Amount**
**Mg stent**	H_3PO_4 (85 wt. %)	37.5% v/v
	EtOH	62.5% v/v
**316L ss stent**	H_3PO_4 (85 wt. %)	42 wt. %
	Glycerol	47 wt. %
	H_2O	11 wt. %

Supplementary Table 3 Parameters used in the electrochemical polishing process for different tracheal stents.

	**Anodic current (A)**	**Applied voltage (V)**	**Time (min)**	**Temperature (°C)**
**LZ61-KBMS stent**	0.6	20-30	4	Dry ice cooling
**AZ31 stent**	0.6	20-30	5	Dry ice cooling
**316L SS stent**	1.2	25-30	3	Tap water cooling
